# Effect of respiratory training on swallowing function in swallowing disorders: a systematic review and meta-analysis

**DOI:** 10.1007/s00405-023-08280-7

**Published:** 2023-10-16

**Authors:** Yinuo Dai, Jianzheng Cai, Haifang Wang, Yingying Zhang, Chunyan Niu, Yalan Wang

**Affiliations:** Department of Nursing, The First Affiliated Hospital of Soochow University, Suzhou, 215006 China

**Keywords:** Breathing exercises, Respiratory muscles, Deglutition disorders, Rehabilitation, Meta-analysis

## Abstract

**Purpose:**

To determine the clinical efficacy of different respiratory training interventions on swallowing function in patients with swallowing disorders through the systematic review.

**Methods:**

We reviewed the literature regarding the application of respiratory training therapy in patients with swallowing disorders, followed by a PRISMA search of published literature in five databases (PubMed, Web of Science, The Cochrane Library, CINAHL and EMBASE) in December 2022. Two reviewers performed study selection, quality evaluation, and risk of bias, followed by data extraction and detailed analysis.

**Results:**

A total of six randomized controlled studies with a total sample size of 193 cases were included. Respiratory training improved swallowing safety (PAS (*n* = 151, SMD = 0.69, 95% CI − 1.11 to − 0.26, *I*^2^ = 36, *p* < 0.001)) and swallowing efficiency [residual (*n* = 63, SMD = 1.67, 95% CI − 2.26 to − 1.09, *I*^2^ = 23%, *p* < 0.001)] compared to control groups. The results of the qualitative analysis conducted in this study revealed that respiratory training enhanced hyoid bone movement but had no effect on swallowing quality of life.

**Conclusions:**

Respiratory training interventions may improve swallowing safety and efficiency in patients with dysphagia. However, the level of evidence is low, and there is a limited amount of research on the effectiveness and physiology of this intervention to improve swallowing function. In the future, there is a need to expand clinical studies, standardize measurement tools, and improve study protocols.

## Introduction

Dysphagia is usually defined as an impairment of swallowing safety and/or swallowing efficiency [1]. This symptom may occur at all stages of life, and one report estimated the prevalence to be about 20% in the general population [2]. But due to age-related changes in swallowing physiology and factors such as disease, it is more prevalent in adults over 65 years of age and in patients with neurological diseases such as stroke, multiple sclerosis and Parkinson’s disease [3, 4]. Dysphagia can reduce quality of life and lead to a number of serious complications, making it a significant source of hospitalization, delayed discharge, and death in the elderly population [5–7]. However, limited by length of stay and medical conditions, current treatment strategies for dysphagia are mainly short-term, and the effectiveness and long-term benefits of most traditional strategies have not been fully elucidated [8–10]. Therefore, it is important to identify a training method that can prolong the benefits of treatment and be optimally effective.

Respiration and swallowing require the activation of common anatomical structures, and respiratory muscle training is based on the principle whereby when the fibers of the respiratory muscles are overloaded, they respond to training stimuli by adapting their structure in the same way as any other skeletal muscle [7, 11]. Previous studies have indicated that inspiratory muscle training (IMT) increases muscle strength and improves muscle function, while expiratory muscle strength training (EMST) results in improved the movement of the neurologically innervated submental muscle complex [12, 13]. In this type of training, patients are asked to perform repetitive breathing with external loads using flow-dependent resistance or pressure threshold tools [14]. There is compelling and high-quality evidence that respiratory training may be an effective non-pharmacological treatment for dysphagia [15].

Current research has confirmed the benefits of respiratory training on respiratory function [16], and also reduced the risk of respiratory complications after stroke [17]. However, most of the available studies report mixed evidence regarding the evaluation of respiratory training on swallowing function, with many studies focusing on the safety of swallowing but lacking in improving swallowing efficiency and related physiological aspects [18, 19], as well as studies evaluating the effects of only one of the training methods and lacking relevance to patients with swallowing disorders [20, 21]. Therefore, we reviewed the available evidence on the effects of multiple respiratory training interventions on swallowing function in patients with swallowing disorders. The main research focus was on the assessment and impact of respiratory training methods on swallowing safety and efficacy. Our research questions were as follows:

1. What are the characteristics of respiratory training interventions used to improve dysphagia?
2. Does respiratory training improve swallowing function (swallowing safety and effectiveness)?

## Methods

We used the Systematic Reviews and Meta-Analyses (PRISMA) statement as guidelines for the development and methodology of this systematic evaluation [22].

### Search strategy

Extensive literature searches were conducted independently by two researchers using PubMed, Web of Science, The Cochrane Library, CINAHL, and EMBASE from the creation of each database through December 2022 by using the following English descriptors: “breathing exercises”, “respiratory muscle training”, “Oropharyngeal muscle strength training”, “inspiratory muscle strength training”, “dysphagia”, “deglutition”, “swallowing disorders” and “oropharyngeal dysphagia”.

### Inclusion and exclusion criteria

Studies that met the following PICOS criteria were included: (1) Participants (P): all study participants were adults who were assessed as having dysphagia by objective instrumental examination; (2) Types of intervention (I): relevant Respiratory training intervention methods were implemented: IMT, EMST, or inspiratory/expiratory muscle training (IEMT); (3) Types of comparisons (C): zero resistance training with dummy equipment, conventional training, or standard training; (4) outcomes (O): valid and reliable outcome measurement methods were used to evaluate the effect of breathing training on swallowing function after intervention; (5) Types of studies (S): randomized controlled trials (RCTs). We restricted the search to English-language publications, excluding studies for which full text was not available. Furthermore, because there are few relevant clinical trials of respiratory training applied to patients with swallowing disorders, we did not restrict the study duration. To improve the quality of included the studies, only RCTs were included in this systematic evaluation, and types of literature such as reviews, case reports, and conference papers were excluded. Since we aimed to study the effect of breathing training on swallowing function in adults with dysphagia, studies with children and animals were also excluded.

### Study selection

**Fig. 1:**
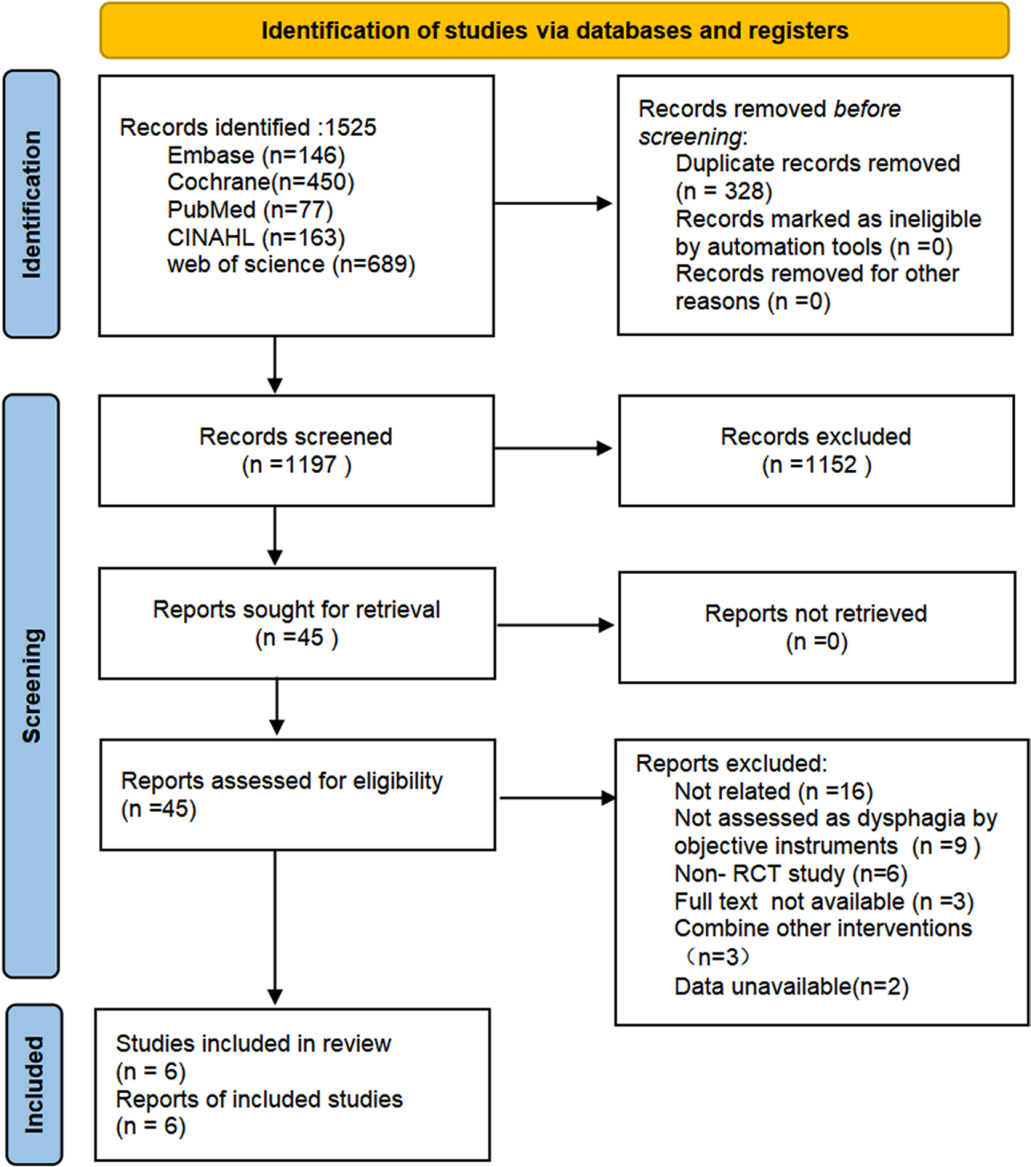
Flow diagram for article inclusion PRISMA

As shown in Fig. 1, a total of 1525 records were obtained from the original search, 328 of which were duplicate records. After combining the duplicate articles, two reviewers were available to independently screen the headlines and abstracts of all retrieved records and determine whether they met the inclusion criteria. Potentially eligible studies were then reviewed in their entirety. The two reviewers were free of potential bias, and differences in study selection were agreed upon through consultation.

### Risk of bias assessment

The methodological quality of the included articles was independently assessed by two reviewers. To assess the risk of bias in RCTs, we used the Cochrane risk of bias tool, rob2.0 [23]. The assessment tool included five evaluation domains (bias during randomization, bias for deviation from established interventions, bias for missing outcome data, bias for outcome measures, and bias for selective reporting of outcomes).

### Data extraction process

Data extraction for articles that met the inclusion criteria was performed independently by two reviewers. The extracted data included the following characteristics: (1) authors and year of publication; (2) study design; (3) participants (number of participants, age, gender, etiology, dysphagia assessment tools); (4) male-to-female ratio of sample size; (5) respiratory training intervention protocol (number of repetitions, frequency, duration); (6) details of the implemented intervention; (7) Interventions in the control group; (8) outcome measures; (9) summary of outcomes and effect sizes. The results of each study were further extracted, including any statistical analysis of changes in swallowing function after the intervention. When some important data were missing, we tried to contact the corresponding author.

### Statistical analysis and grading the evidence

Study data were combined if the outcome measures used in at least two studies were comparable. A meta-analysis was designed using Review Manager (version 5.4) according to the practice recommendations of the Cochrane Handbook.

The included studies were assessed using chi-square tests and I^2^. The variables of interest included in this study were continuous, and for *I*^2^ < 50, we prioritized a fixed effects analysis model to calculate the mean difference (MD) and 95% confidence intervals (CI). According to Cochrane’s recommendations, studies reporting median, quartile, and range of data were not included in the meta-analysis because we lacked accurate underlying data, which would have skewed results [24]. If *I*^2^ > 50% was considered that significant heterogeneity was observed, and thus we analyzed the source of heterogeneity by subgroup analysis and sensitivity analysis using the random effects model. Subgroup analysis is planned for different types of respiratory training interventions. For the assessment of Penetration Aspiration Scale (PAS) scores, residuals, we tested for differences using 95% CIs and standardized mean difference (SMD) to explain whether the different assessment methods affected the results.

The Grading of Recommendations, Assessment, Development and Evaluations tool (GRADE) [25] was utilized in this study to provide a quality rating of the evidence and thus judge the credibility of the results. Due to the number of studies in the meta-analysis being less than 10, we did not assess reporting bias.

## Results

### Literature retrieval

Figure 1 reports the selection process for the included studies. Of the 1525 studies that were available for initial screening, we excluded 1480 that were duplicates and not relevant to this study. As such, 45 articles that potentially met the relevant study criteria were screened and evaluated in full text, with particular attention to factors such as study design and intervention (type of treatment and outcome evaluation metrics). Subsequently, 16 full-text articles were excluded because they did not mention respiratory training interventions for dysphagia; a further nine articles were studied in patients who were assessed as having dysphagia without objective instrumentation; six articles were non-RCTs; three articles had additional combinations of interventions in addition to breathing training; and two articles lacked available data. Overall, six articles met the purpose and inclusion criteria of this review.

### Assessment of risk of bias

**Fig. 2:**
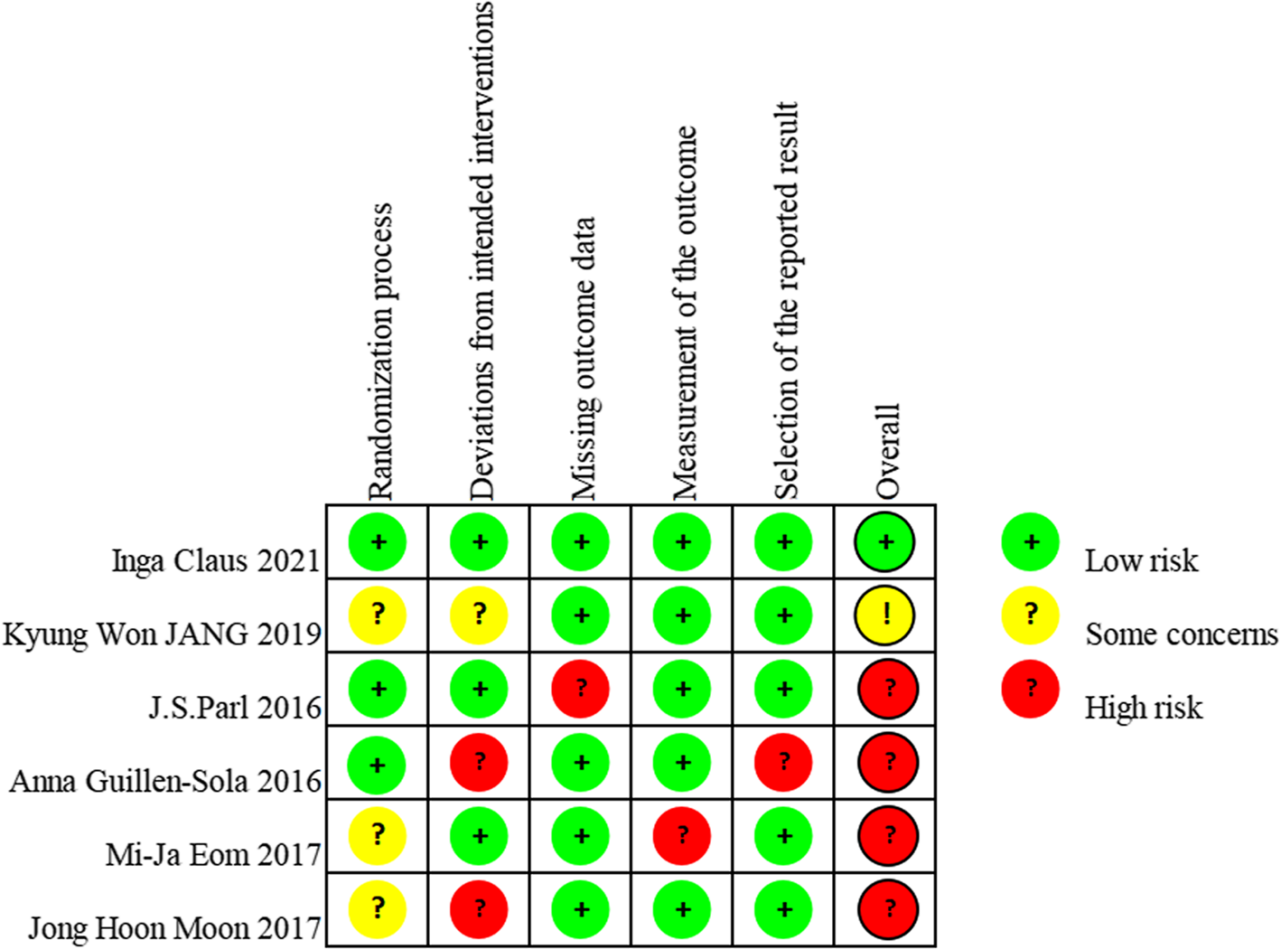
Risk of bias assessment of included studies

Figure 2 summarizes the risk of bias of all included RCTs using the Cochrane risk of bias tool rob2.0. Almost none of the included studies mentioned distribution concealment. Only one study, with a sample size of 45 patients with Parkinson’s disease, was associated with a low risk of bias in all regions [15]. All other studies exhibited a medium or high risk of bias in at least one domain. The risk of bias in the included studies was primarily associated with a lack of allocation concealment, inadequate blinding of the study process, and/or the assessment of the impact of lost access data on the study.

### Assessment of the quality of the evidence

**Fig. 3:**
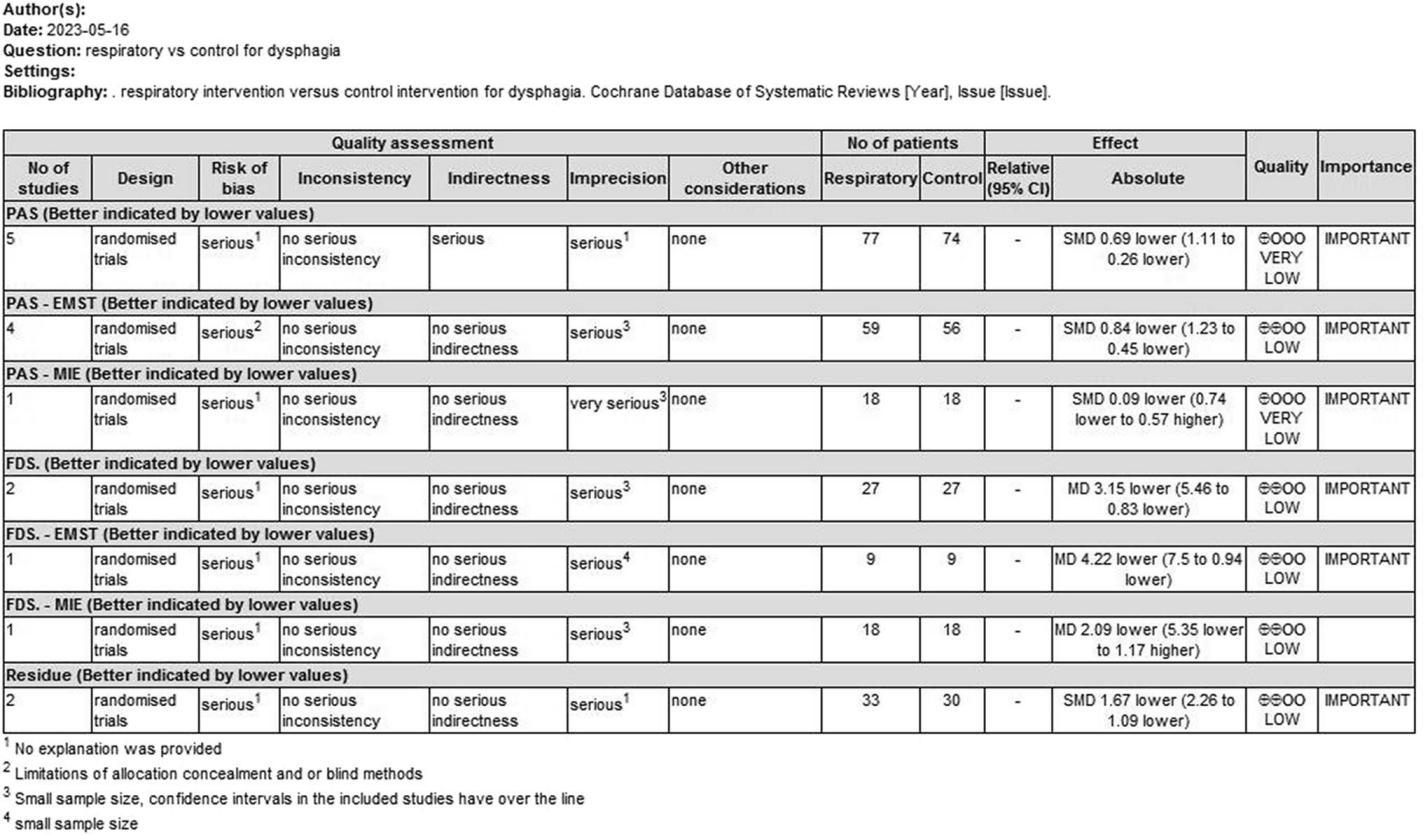
(GRADE) tool assessment of the quality of the evidence

Evaluation of the quality of evidence ratings using GRADE revealed that the ratings were all low for included studies; this finding was most often a result of inadequate blinding of the study design and/or a small sample size (Fig. 3).

### Subgroup and sensitivity analysis

The data can be used to complete subgroup analyses of different respiratory training modalities: swallowing safety (PAS) and swallowing function (FDS). Due to the small number of relevant studies and the fact that the included studies were considered to have a high risk of bias, no sensitivity analysis was conducted.

### Question 1: Patient and intervention characteristics

The patient and intervention characteristics are described in Table 1. Of the included studies, the sample size ranged from 18 participants [28] to 45 participants [15], with a total sample size of 193 cases. The participants included in the studies were generally of old age, with a larger proportion of males included overall. Two different groups of patients were included in this study: stroke [26–30] and Parkinson’s disease [15], all of whom were diagnosed with varying degrees of dysphagia. Currently, respiratory training in patients with dysphagia is primarily focused on the expiratory muscles. All four of the included studies included EMST [15, 27, 29, 30]. The dysphagia population assessment instrument for one of the included studies was the Flexible Endoscopic Evaluation of Swallowing (FEES), and all other studies used the Video Fluoroscopic Swallowing Study (VFSS) for objective assessment. One study implemented IEMT [28], and one study employed Mechanical Inspiration and Expiration (MIE) [26]. In the four studies [15, 27, 28, 30], the EMST150 was the handheld device used for respiratory training, and another study used a similar but different model of respiratory training device [28]. All studies provided information on whether to implement and adjust the load depending on the patient’s individual situation. The duration of treatment utilized in the regular training program was 2–4 weeks, with a frequency of 5 or 7 days of training per week and 5 sets of training per day with several breaths per set. Only one study differed in its use of mechanical devices and a training protocol of 30 min per day [26]. The control group received zero resistance training with dummy equipment, conventional training, or standard training. Notably, we observed in one study that patients completed the training independently at home under various forms of clinical guidance from professional therapists or rehabilitators [15] (see Table 1).

**Table 1:** Patient and intervention characteristics

Study	Year	*N* (M/F)	Average age in years (SD)	Etiology	Dysphagia assessment tools	Parameters	Control group	Study design	Guidance
Inga Claus et al. [15]	2021	TG24 (19,5) CG21 (18,3)	TG 67.3 (9.5) CG 67.1 (7.7)	Parkinson	FEES	Resistance: 75% MEP Progression: fixed	Sham device exercise	Prospective, double-blind, randomized, controlled trial	Introduced during the first study visit and a telephonic evaluation was performed during the training period
Kyung Won Jang et al. [26]	2019	TG18 (10,8) CG18 (9, 9)	TG67.28 (9.48) CG71.15 (8.61)	Stroke	VFSS	Starting: inspiration, positive pressure was 15–20cmH_2_O; expiration, similar to the inspiration pressure; Progression: inspiration, increasing to 40 cmH_2_O; expiration, increased to 10–20 cm H_2_O	Traditional therapy	Prospective, randomized, controlled trial	Two experienced physiatrists (no home practice)
J.S. Parl et al. [27]	2016	TG14 (6,8) CG13 (6,7)	TG 64.3 (10.7) CG 65.8 (11.3)	Stroke	VFSS	Resistance: 70% MEP Progression: fixed	Sham device exercise	Prospective, single-blind, randomized, controlled trial	Direct supervision by therapists (no home practice)
Anna Guillén-Solà et al.[28]	2016	TG20 (16,4) CG21 (12,9)	TG 67.9 (10.6) CG 68.9 (7.0)	Stroke	VFSS	Resistance: 30%MIP + 30%MEP Progression: increased 10 cmH_2_O weekly	Standard swallow therapy	Prospective, single-blind, randomized-controlled trial	Direct supervision by therapists (no home practice)
Mi-Ja Eom et al.[29]	2017	TG13 (5, 8) CG13 (6, 7)	TG 69.2 (4.1) CG70.2 (3.6)	Stroke	VFSS	Resistance: 70% MEP Progression: fixed	Sham device exercise	Prospective, randomized, controlled trial	Direct supervision by an experienced physician and occupational therapist (no home practice)
Jong Hoon Moon et al. [30]	2017	TG9 (6, 3) CG9 (6, 3)	TG 63.0 (5.8) CG 63.1 (5.2)	Stroke	VFSS	Resistance:70% MEP Progression: fixed	Traditional therapy	Prospective, randomized, controlled trial	Direct supervision by therapists (no home practice)

*N* sample size, *M* male, *F* female, *TG* treated group, *CG* control group, *SD* standard deviation, *EMST* expiratory muscle strength training, *IEMT* inspiratory/expiratory muscle training, *MIE* mechanical inspiration and expiration, *MEP* maximal expiratory pressures, *MIP* maximal inspiratory pressures

### Question 2 respiratory training intervention results

#### Swallowing safety

**Fig. 4:**
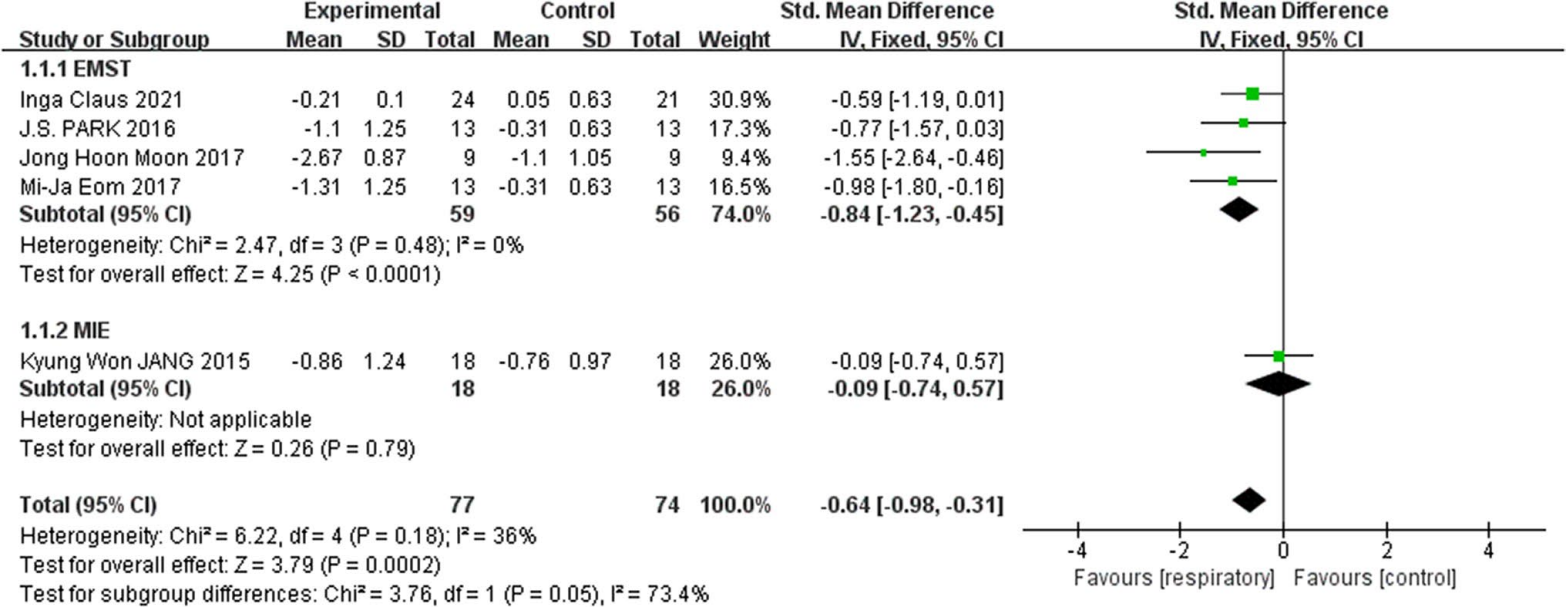
Respiratory training versus control group: Penetration Aspiration Scale

The studies included in the quantitative analysis ubiquitously assessed the effect of applied respiratory training interventions upon swallowing safety based on the PAS. One IEMT study did not have complete data after respiratory intervention and only included changes in the number of people with PAS > 5 and PAS < 5 after intervention without statistical significance [28]. Therefore, a total of five studies were included for quantitative analysis [15, 26, 27, 29, 30]. Because one of the studies [15] was assessed using different scoring levels, and the results of a meta-analysis with continuous outcomes revealed heterogeneity across studies, it was more scientifically valid for us to use SMD as an effect size. Overall, there was evidence of a statistically significant improvement in swallowing safety associated with breathing training compared to the no or sham breathing intervention groups: PAS scores were reduced by 0.69 (*n* = 151, 95% CI − 1.11 to − 0.26, *I*^2^ = 36, *p* < 0.001). However, there was a significant subgroup interaction (*p* = 0.05, *I*^2^ = 73.4%) in the subgroup analysis of intervention types, and EMST was statistically significant, but MIE training showed no statistically significant reduction in PAS scores compared with the control group (*p* = 0.79) (Fig. 4).

**Fig. 5:**
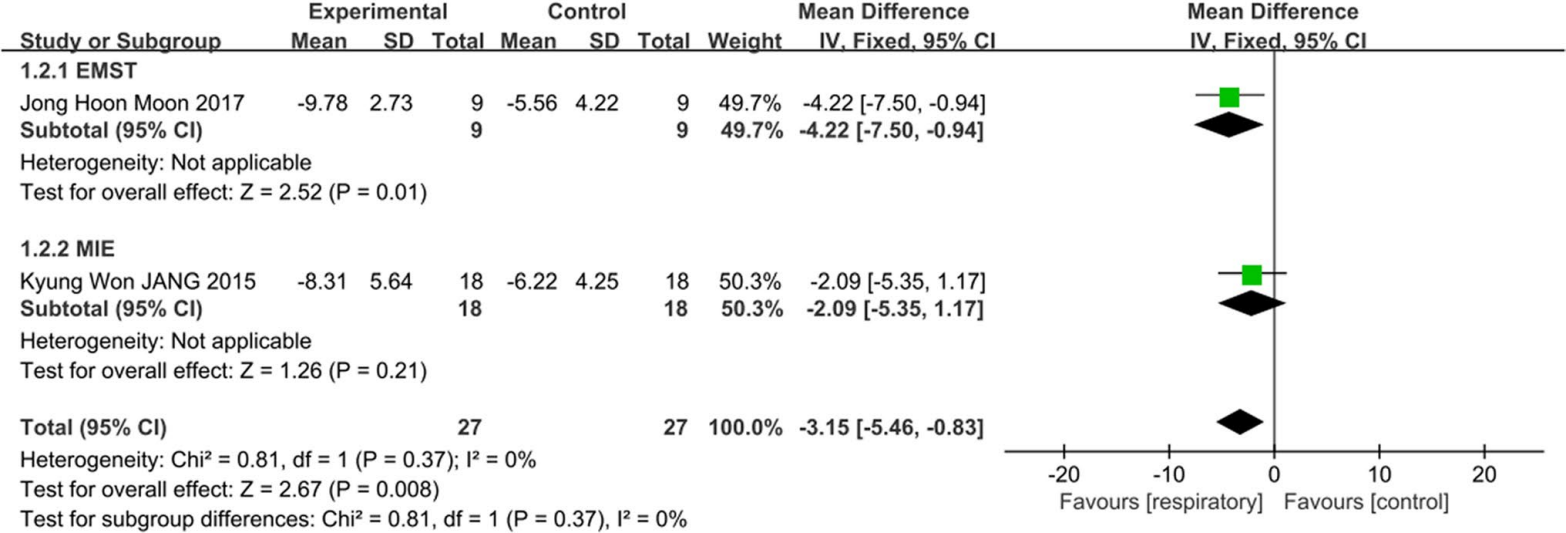
Respiratory training versus control group: Functional Dysphagia Scale

Combining data from two study outcomes [26, 30], respiratory training was revealed to improve swallowing function (FDS, *n* = 54, MD = − 3.15, 95% CI − 5.46 to − 0.83, *I*^2^ = 0, *p* < 0.001). Subgroup analysis of MIE and EMST interventions showed a significant improvement in EMST (*n* = 18, MD = − 4.22, 95% CI − 7.50 to − 0.94, *p* = 0.01), while the change was not statistically significant in MIE (*n* = 36, MD = − 2.0, 95% CI − 5.35 to 1.17, *p* = 0.21) and no significant subgroup interactions (*p* = 0.37, *I*^2^ = 0%) (Fig. 5). However, improvement in nasal permeation scores was statistically significant in only one of the two studies (*p* = 0.04) [26], while the other study reported a statistically significant improvement in overall FDS scores only.

The results of the Volume Viscosity Swallow Test (VVST) study by Guillén-Solà et al. [28] revealed that after 3 weeks of intervention, patients in the IEMT group exhibited significant improvements in safety signs (*p* = 0.011), but this beneficial effect was not displayed after 3 months of follow-up (*p* = 0.5).

### Swallowing efficiency

#### Swallowing physiology

In one study that included the physiological activity of swallowing as an outcome, breathing exercises were significantly effective in improving swallowing motions. sEMG results revealed a statistically significant increase in suprahyoid muscle activity after respiratory training intervention [27]. The experimental group in one study revealed increased improvement in the oral and pharyngeal stages of Video fluoroscopic Dysphagia Scale than in the control group; the difference between the groups before and after treatment was statistically significant [29]. However, due to inconsistent measurement ranges and distinct measurement tools, it was not possible to integrate the data for reporting.

### Swallowing residue

**Fig. 6:**
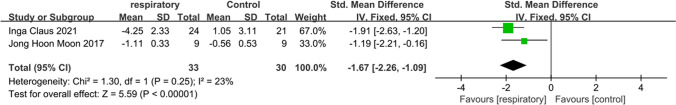
Respiratory training versus control group: residual

Three studies reported results regarding swallowing residue [15, 28, 30], using the VFSS to assess the residual score, or Vallecular Residue (VR). One study implemented the VVST and revealed an improvement in the efficacy signs of the inspiratory mechanism, but it lacked the underlying data and was not included in the study [28]. Our use of SMD as the effect size is because the two studies were evaluated using different scoring levels. Swallowing residual (*n* = 63, SMD = − 1.67, 95% CI = − 2.26 to − 1.09, *I*^2^ = 23%, *p* < 0.001) was identified in a combined meta-analysis (Fig. 6).

### Additional results

Two studies reported significant improvements in overall swallowing function as assessed by the Functional Oral Intake Scale, however, these findings could not be quantitatively analyzed due to a lack of data [27, 28]. Only one study used SWAL—QOL as an outcome measurement and the intervention results showed no statistical significance [15]. Table 2 provides detailed information regarding the measures and results of all reports analyzed in the present study, including the Swallowing Disturbance Questionnaire, quality of life measurements, and so on.

**Table 2:** Swallowing measures and results

Study	Inclusion criteria	Outcome measures collected	Results
Inga Claus et al.[15]	(1) Confirmed pharyngeal dysphagia by FEES; (2) Medication and nutritional therapy (at least 4 weeks prior to enrollment); (3) No other neurological disorders	Overall FEES dysphagia score (premature spillage, PAS, residue) SWAL-QOL/SDQ	No significant differences in premature spillage and PAS; Significant improvement in residue scores in the active group (*p* < 0.01), and at follow-up (*p* < 0.05); No significant intervention effect was observed using the SWAL-QWL system(p = 0.45); significant improvement in SDQ (p < 0.01), as well as a prolonged intervention effect (P < 0.05);
Kyung Won JANG et al.[26]	(1)Diagnosed with VPI (2) No pulmonary diseases; (3) No previous stoke or pharyngeal structural abnormalities; (4) No deteriorated cognitive function or mentality; (5) No medical conditions that could affect their swallowing ability	ASHA-NOMS FDS PAS	No significant differences were observed in the swallowing scores using ASHA-NOMS; Significant improvement observed in the nasal penetration degree of FDS vs the control group (p < 0.05), Statistically significant differences in the total FDS, the oral phase and the pharyngeal phase; No significant changes reported;
J.S. Parl et al.[27]	(1) Diagnosed dysphagia following a stroke (2) Stroke onset less than 6 months and MMSE > 24 (3) No dysphagia before stroke, serious facial problems, unstable breathing and pulse (4) No tracheostomy (5) No severe communication disorder (6) No inadequate lip closure	sEMG PAS FOIS	Significant differences observed in the activity of the suprahyoid muscles between groups (*p* = 0.01) Significant differences reported in activity of the suprahyoid muscles between the groups (*p* < 0.01) Significant differences observed between groups in the liquid type PAS score, but not of the semisolid type (*p* = 0.03, *p* = 0.32) Significant differences were observed after treatment in only the liquid type PAS scores and not the semisolid type in both groups (*p* = 0.03, *p* = 0.38) Significant improvement in both groups (*p* = 0.04), but no statistically significant differences between groups (*p* = 0. 06)
Anna Guillén-Solà et al.[28]	(1) Diagnosed subacute ischemic stroke within 1–3 weeks (2) PAS score⩾ 3 (3) No cognitive impairment (Short Portable Mental Status Questionnaire < 3) (4) No history of previous neurological diseases	PAS FOIS DOSS V-VST (efficacy signs, security signs)	No differences in PAS or respiratory complications were detected between the groups at the 3-month follow-up No significant differences were observed in FOIS and the DOSS between the study groups Significant improvement in efficacy signs at 3 months (*p* = 0.037) and in security signs after 3-weeks (*p* = 0.049). Beneficial effect in security signs was lost at 3-month follow-up
Mi-Ja Eom et al. [29]	(1) Having dysphagia caused by a stroke (2) Age > 65 (3) Onset duration of < 3 months and MMSE ≥ 24 (4) No unstable breathing and pulse (5) No serious facial problems, tracheostomy, severe communication disorder and inadequate lip closure	VDS (oral phase, pharyngeal phase, total score) PAS	Significant improvements reported in both oral and pharyngeal phases of VDS (all p < 0.05). The experimental group exhibited further improvement in the oral and pharyngeal phases of VDS (*p* = 0.042, 0.047), with the differences between groups being statistically significant (*p* < 0.01, 0.038). Significant improvement reported in the experimental group (*p* < 0.01). The difference between the groups was statistically significant (*p* = 0.027)
Jong Hoon Moon et al.[30]	(1) Diagnosed as pharyngeal dysphagia (2) Onset of no more than 1 month (3) No oral stage problems (5) MMSE ≥ 24 (6) No specific medical problems (7) Albescence of appropriate lip closing (8) No significant facial paralysis, tracheostomy and percutaneous endoscopic gastrostomy, and hypertension	FDS PAS VR PR	Significant improvement was observed in all variables (*p* < 0.05), apart from in PR in the control group. The experimental group exhibited great improvement versus the control group in FDS, VR and PAS (*p* < 0.05)

*FEES* flexible endoscopic evaluation of swallowing, *PAS* penetration-aspiration scale, *SWAL-QOL* swallowing quality of life questionnaire, *SDQ* swallowing disturbance questionnaire, *VPI* velopharyngeal incompetence, *ASHA-NOMS* American speech-language-hearing association’s national outcome measurement system, *FDS* functional dysphagia scale, *sEMG* surface electromyography, *MMSE* mini–mental state examination, *FOIS* functional outcome intake scale, *DOSS* dysphagia outcome severity scale, *VVST* volume viscosity swallow test, *VDS video* fluoroscopic dysphagia scale, *VR* vallecular residue, *PR* Pyriform sinuses residue

## Discussion

Swallowing function is closely associated with respiratory function [29]. Previous studies have confirmed that exercising respiratory muscles by applying mechanical loads could effectively improve the ability to clear the airway and ameliorate cough function [31]. Therefore, respiratory training for patients with dysphagia is necessary and an important measure to prevent aspiration. One study revealed by sEMG that the activity of the supraglottic tissue group was significantly higher in the experimental group than in the control group after a respiratory training intervention [27]. Stimulating supraglottic activity thus was observed to have an effect on hyoid elevation and upper esophageal sphincter opening. These findings suggest that respiratory training may enhance muscle activity and improve coordination between respiratory and swallowing muscles to improve overall swallowing function [32, 33].

Therefore, we systematically reviewed the efficacy of breathing training interventions to improve swallowing function. Four of the six studies included expiratory muscle exercises, one mechanical inspiratory expiratory exercise, and one inspiratory expiratory exercise. The results of the MIE subgroup analysis show that there are too few included studies, a small sample size, and a wide confidence interval. The included IEMT did not have enough data available to merge results. Therefore, we cannot determine the effect of these two training interventions on swallowing function. In the study of stroke and Parkinson’s disease patients, EMST can effectively reduce aspiration and residual so as to improve the safety and efficiency of swallowing.

The ability to improve the safety of swallowing is important in effectively reducing or minimizing serious complications in patients with dysphagia. This meta-analysis revealed that RCT data from the five included studies reported statistically significant overall reductions in scores after respiratory training based on PAS, but subgroup analysis showed a nonsignificant improvement in MIE. Moreover, FDS scores were reported to be significantly improved in the two included studies, and one of the studies indicated a significant improvement in nasal penetration, an effect that could still be observed during later follow-ups [26]. Overall, these data suggest that respiratory muscle training can improve swallowing safety. In a review of all included articles, the results of one study indicated a significant difference in liquid-type PAS scores between groups, while the difference in semisolid-type PAS scores was not statistically significant [27]. In contrast, most trials did not specify which type of push injection was used to assess the training effect. This may be one of the reasons for the differences in outcomes during the assessment of swallowing safety. Secondly, the average duration of current respiratory training interventions is around 4 weeks. This systematic review includes only two studies assessing the impact of respiratory training after the cessation of such training, and any changes that occur afterwards [15, 28]. Thus, it is unclear whether extended maintenance treatment (possibly in the form of a facilitated course of treatment) is required. Moreover, we therefore suggest future studies are required that use more precise and uniform methodological quality assessment methods and extended treatment follow up to better assess improvements in safety associated with respiratory training in patients with dysphagia.

Swallowing efficiency evaluation is an important indicator of intervention outcome, which primarily includes analysis of swallowing physiology and residual analysis after swallowing [34, 35]. The meta-analysis of the control studies revealed a positive effect of EMST on improving residuals, but cautious interpretation of these results is required due to a lack of focus on improving swallowing efficiency in the current studies and the heterogeneity of the assessment tools. The physiology of swallowing is another aspect that responds to swallowing efficiency. In the included studies, we only observed physiological alterations in patients receiving EMST training. One study detected enhanced supraglottic activity by surface electromyography, which demonstrates that swallowing-related muscles can be employed as a complement to motor units during respiratory training [28].

Across all respiratory training intervention studies included in the present meta-analysis, we observed heterogeneity in the objective assessment instruments, training protocols, and outcome measurement tools. As such, it was important to select rigorous and objective assessment and outcome measures. The FEES analysis and the VFSS are commonly chosen in the clinic to assess and examine swallowing function. However, these quantitative assessments utilizing video fluoroscopy remain largely subjective in assessing swallowing ability and in obtaining information regarding the physiology of swallowing in patients [36], and the method has limitations in terms of the population to which it can be applied, making it difficult to perform repeat operations in a short period of time. sEMG facilitates the objective and detailed recording of data used to assess the magnitude and duration of different swallowing events and the changes in strength of each muscle involved in oropharyngeal swallowing without further exploring changes in swallowing physiology resulting from respiratory training [37]. As such, sEMG provides a scientific basis for determining the optimal breathing training program for individual patients based on the physiological characteristics of swallowing in different patients, thus it can be considered for widespread clinical use in future studies [38, 39]. In the meantime, we observed that current respiratory training techniques are dominated by expiratory muscle training. It has been suggested that combined inspiratory and expiratory muscle training may better improve airway safety in swallowing [40, 41]. Although this hypothesis requires validation by future studies that should continue to explore the effects of different modes of breathing training on dysphagia in order to identify the appropriate training mode for the patient.

Only one of the included studies evaluated the effect of respiratory training on the quality of life of patients with swallowing disorders [15]. The studies did not identify statistically significant differences in overall scores compared to traditional methods or sham groups, but patients’ self-perceived respiratory training improved dysphagia symptoms. However, previous studies did report that respiratory training can significantly improve the burden and mental health of patients with swallowing disorders [42]. In future studies, the inclusion of patients’ self-perceptions could be considered an important part of the assessment.

To date, no systematic review has evaluated respiratory training interventions in patients who have been diagnosed with swallowing disorders by objective instrumentation. The results of our review were compared with recent reviews on the effects of EMST interventions on swallowing function [20, 21]. These reviews highlighted the heterogeneity of existing research evaluation tools, the small sample sizes of the studies and methods, and the low quality of research. However, this study evaluated multiple respiratory training interventions, and our included RCTs provided conclusions about the effectiveness of respiratory training on swallowing function. We have a higher quality of evidence under the scientific guidance of the Cochrane method. The results suggest that respiratory training can be considered for its safety and efficacy in the clinical improvement of dysphagia.

## Limitations

The limitations of this review are associated with the limitations of the articles selected for analysis. Most of the literature utilized small sample sizes and poor-quality study designs, resulting in low levels of evidence for the resulting conclusions. Secondly, we excluded unpublished articles from the literature search and included only English-language studies. This contributed to the inclusion of fewer articles. When screening the full text, we exclusively included studies in which patients were diagnosed with dysphagia by objective instrumentation, which resulted in patients with undiagnosed mild dysphagia groups or diagnosed with other methods not being included in studies of observation of the effects of intervention.

## Conclusions

In conclusion, this systematic review and meta-analysis analyses the effects of breathing training on patients with swallowing disorders. EMST was overall identified as being effectively utilized to improve swallowing safety and residual in patients with swallowing disorders, but the evidence is limited. We further observed an improvement effect of respiratory training upon swallowing activity with increased hyoid movement but did not, however, identify a significant improvement in quality of life. This field requires further extensive clinical studies using objective, rigorous methodological tools to quantify the physiological effectiveness of respiratory training to better individualize training programs for patients with dysphagia.

## Data Availability

The data that support the findings of this study are available from the corresponding author upon reasonable request.
